# Evaluation of Plasma MicroRNAs as Diagnostic and Prognostic Biomarkers in Pancreatic Adenocarcinoma: miR-196a and miR-210 Could Be Negative and Positive Prognostic Markers, Respectively

**DOI:** 10.1155/2017/6495867

**Published:** 2017-03-29

**Authors:** Qi Yu, Changqing Xu, Wei Yuan, Chengfeng Wang, Ping Zhao, Lianzhen Chen, Jie Ma

**Affiliations:** ^1^Department of General Surgery, Beijing Rehabilitation Hospital, Capital Medical University, Beijing 100044, China; ^2^Department of Digestive Medicine, Qianfoshan Hospital, Shandong University, 16766 Jingshi Road, Jinan 250014, China; ^3^State Key Laboratory of Molecular Oncology, National Cancer Center/Cancer Hospital, Chinese Academy of Medical Sciences and Peking Union Medical College, Beijing 100021, China; ^4^Department of Abdominal Surgery, Cancer Hospital of Chinese Academy of Medical Sciences, Peking Union Medical College, Beijing, China; ^5^Beijing Hospital, National Center of Gerontology, Beijing 100730, China

## Abstract

*Background*. Identifying diagnostic and prognostic biomarkers that could be targeted in the therapy of pancreatic cancer is essential.* Objective*. Investigations were conducted with respect to plasma miRNA (miR-21, miR-210, miR-155, miR-196a, miR-20a, and miR-25) expression and clinicopathologic factors to evaluate the prognostic value of miRNAs in pancreatic ductal adenocarcinoma (PDAC).* Methods*. Plasma miRNAs were detected by real-time quantitative PCR, and the association with clinicopathologic factors was subsequently performed by univariate and multivariate analyses.* Results*. Six miRNAs expressed significantly higher in PDAC patients than in normal individuals were identified. Receiver operating characteristic (ROC) curves were constructed. It was evident that miRNA expression associated with PDAC, lymph node metastasis, serosal infiltration, and comprehensive therapy reached significance for overall survival. High miR-196a expression was associated with poor survival (*P* = 0.001), whereas high miR-210 expression was significantly associated with improved survival (*P* = 0.003). Multivariate survival analysis indicated that the miR-210 and miR-196a expression signature, lymph node metastasis, and comprehensive therapy were independent factors affecting overall survival.* Conclusions*. MiRNA expression profile is distinctive in PDAC. Aberrant expression of certain miRNAs was remarkably involved in shaping the overall survival time, which include miR-196a overexpression and decreased miR-210 expression.

## 1. Introduction

Pancreatic cancer is one of the most fatal cancers worldwide, and it has been a therapeutic challenge for years, due to difficulties and delays in diagnosis, which results in poor prognosis. Pancreatic cancer ranks the fourth leading cause of cancer-related deaths in America [1] and the sixth in China [2]. Poor diagnosis results due to the relatively special anatomical position of the pancreas, which is situated deeply and adjacent to the superior mesenteric vein and artery. Also, the patients in the early stages of pancreatic cancer do not present specific symptoms. Moreover, the radical resection rate of this malignancy is only 10%–15% [3]. Despite surgical resection, the median overall survival in patients with advanced disease stage is about 6 months [4].

During the last decade, the prognosis of pancreatic cancer has not showed any remarkable improvements regardless of the progress in surgical techniques and diagnostic methods. Hence, it is vital to identify distinct in vivo markers for pancreatic cancer in terms of diagnosis and prognosis. This may help to improve the success rates of radical resection and assist in decisions during therapeutic protocols.

The aberrant expression of several miRNAs, considered as diagnostic markers of malignancies, has been widely reported. The overexpression and underexpression of miRNAs have been detected in resected pancreatic cancer tissue specimens, some of which have been evaluated as prognostic markers [5–8]. It was revealed that miRNAs were stable in the plasma and showed the potential to serve as diagnostic and/or prognostic biomarkers. Earlier report about plasma miRNA profile in patients with pancreatic cancer, by Wang et al. [9], indicated that four miRNAs (miR-21, miR-210, miR-155, and miR-196a) were found to be highly expressed in pancreatic cancer tissues, as well as in the plasma. The four miRNAs had a sensitivity of 64% and a specificity of 89% for pancreatic cancer. Subsequently, however, Liu et al. [10] reported 6 new miRNA-based biomarkers (miR-20a, miR-24, miR-25, miR-99a, miR-185, and miR-191) along with miR-21 and indicated that they were highly sensitive and specific in distinguishing various stages of PDAC from cancer-free pancreatic tissues. In addition, miR-21 expression levels in the serum were substantially associated with overall survival. On analyzing the diagnostic value of miR-21, miR-155, and miR-196a, Kong et al. [11] suggested that serum miR-196a could be a potential marker for PDAC prognosis.

Considering that as most studies used tissues resected from patients undergoing surgery, we evaluate the association between the plasma miRNA expression profiles in the resected tissues with the overall survival of PDAC patients. This may help in the decision-making process of the therapeutic method in PDAC patients. In this study, six known miRNAs (miR-21, miR-210, miR-155, miR-196a, miR-20a, and miR-25) whose diagnostic value was previously reported were selected for further examination of their prognostic values in PDAC.

## 2. Materials and Methods

### 2.1. Experimental Subjects

Our study recruited 31 PDAC patients from the Department of abdominal surgery of the Cancer Hospital and Institute of the Chinese Academy of Medical Sciences (Beijing, China) between September 2012 and September 2013, who were confirmed by either pathological examination or fine-needle aspiration cytology (during surgery or by EUS). Twenty-eight healthy individuals were taken up in the study as control, who are all confirmed with healthy conditions after undergoing blood tests and imaging examinations in the Department of Cancer Prevention. This study was conducted in conformity to The Code of Ethics of the World Medical Association (Declaration of Helsinki), printed in the British Medical Journal (18 July, 1964).

Among the 31 PDAC patients, 20 (64.5%) were male and 11 (35.5%) were female, and the age ranged from 47 to 73 (48.82 ± 9.75) years. The tumor in 26 patients (83.9%) was in the head and neck region of the pancreas and in 5 patients (16.1%) in the pancreas body and tail. Nine patients (29.1%) had radical surgery, achieving R0 resection. Seventeen patients (54.8%) received palliative surgery. The remaining 5 patients (16.1%) did not undergo any interventions for tumor metastasis or for other reasons. Comprehensive therapy includes postoperative chemotherapy, radiotherapy, chemoradiotherapy, or intraoperative radiotherapy. Distant liver, lung, or mesenteric dissemination, peritoneal seeding, and supraclavicular lymph node metastases were evaluated before the surgery.

For the patients receiving radical surgery, several characteristics which include tumor size, lymph node metastasis, and serosal or vessel infiltration were evaluated based on the pathological examination. In the patients who received palliative surgery and in those who were unsuitable for surgical treatment, these characteristics were assessed by endoscopic ultrasonography, dynamic computed tomography, or magnetic resonance imaging.

Demographic data for PDAC patients and healthy controls are listed in Table 1. No significant differences in terms of gender, age, cigarette smoking, and alcohol consumption between the two groups (*P* > 0.05) were observed. Clinical characteristics of the PDAC patients are detailed in Table 3.

### 2.2. Plasma Collection

Blood samples were collected from individuals in the two groups before surgery or other therapies and centrifuged at 1,200*g* at 4°C for 10 min to obtain the plasma for miRNA detection. The plasma was then further centrifuged at 1,2000*g* at 4°C for 10 min to eliminate larger molecules. Each plasma sample (200 *μ*L at least) was transferred into 1.5 mL Eppendorf tubes and stored at −80°C for further total RNA isolation.

### 2.3. RNA Extraction

Plasma samples were thawed on ice and 200 *μ*L was transferred into a tube containing 1000 *μ*L Qiazol lysis reagent (Qiagen, Germany). After vortexing, the tube containing the lysate was left at room temperature (15–25°C) for 5 min. Subsequently, 3.5 *μ*L miRNeasy Serum/Plasma Spike-In Control (1.6 × 10^8^ copies/*μ*L working solution, miR-39) (Qiagen, Germany) was added to the tube and samples were mixed thoroughly. RNA was isolated by using miRNeasy Serum/Plasma Kit (Qiagen, Germany) following the manufacture's protocol. Each 12 *μ*L RNA pellet was placed in the RNase-free collection tube and stored at −80°C.

### 2.4. Absolute Quantification of Plasma miRNA

Briefly, miRNeasy Serum/Plasma Spike-In Control (miR-39) and 2 *μ*L of plasma-derived total RNA were reversely transcribed according to the Reverse Transcription Reaction Kit protocol. cDNA from miR-39 was diluted to different concentrations following the cDNA serial dilution protocol. All cDNAs were prepared for real-time PCR according to miScript SYBR Green PCR Kit (Qiagen, Germany) protocol. The mean CT values of miRNeasy Serum/Plasma Spike-In Control (miR-39) and samples from each reaction were calculated by using the Roche® LightCycler® 480 software. The standard curves were generated by plotting the log copy number and CT values. The recovery was determined based on absolute quantification (copies/*μ*L) for each sample.

### 2.5. Statistical Analysis

To employ the SPSS 17.0 software, the Chi-square test was used to examine the differences in the demographic data between the two groups. Quantification of miRNAs' expression in the two groups was compared by Student's *t*-test (SPSS 17.0 software). Receiver operating characteristic curves (ROC curves) were established to further confirm miRNA expression in PDAC diagnosis. For the univariate survival analysis of conventional prognostic factors, the analysis of median survival time of postoperative survival between the two groups was evaluated based on clinicopathological or miRNAs expression' level in terms of the Kaplan-Meier method and log-rank analysis. Variables with significant *P* values from the univariate analysis were taken into the multivariate analysis by using Cox regression. Only *P* value of less than 0.05 (two-sided) was defined as statistically significant.

## 3. Results

### 3.1. miR-21, miR-210, miR-155, miR-196a, miR-20a, and miR-25 Have Strong Diagnostic Value of PDAC Patients

**Figure 1:**
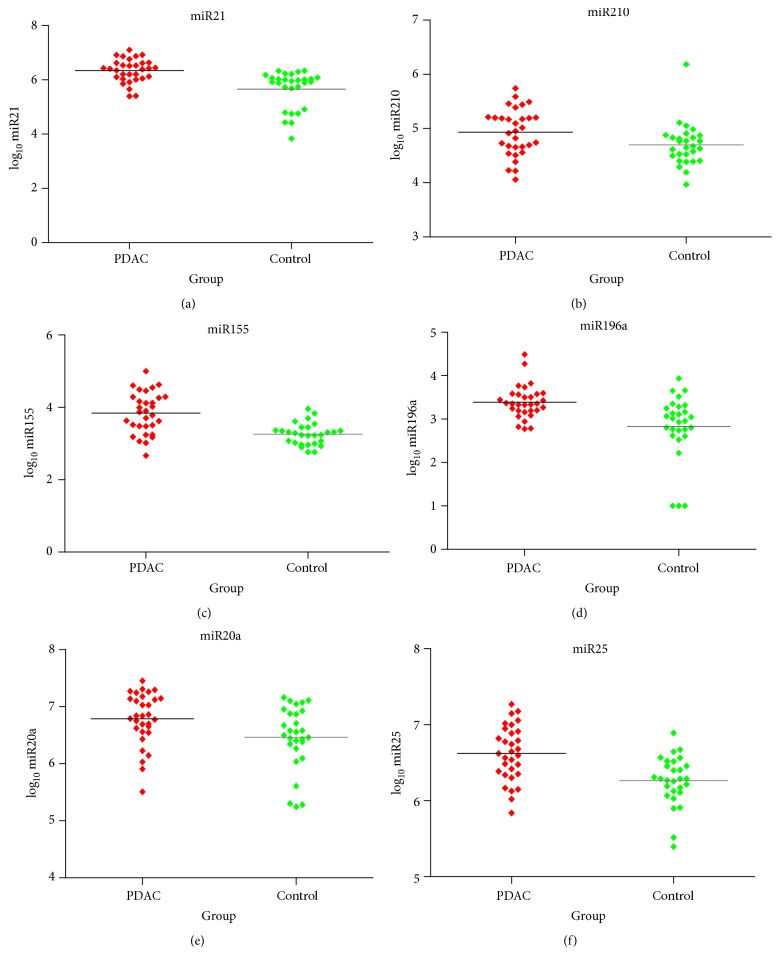
Expression levels for the six miRNAs in the plasma from PDAC patients and control individuals. (a)–(f) represent miR-21, miR-210, miR-155, miR-196a, miR-20a, and miR-25.

**Figure 2:**
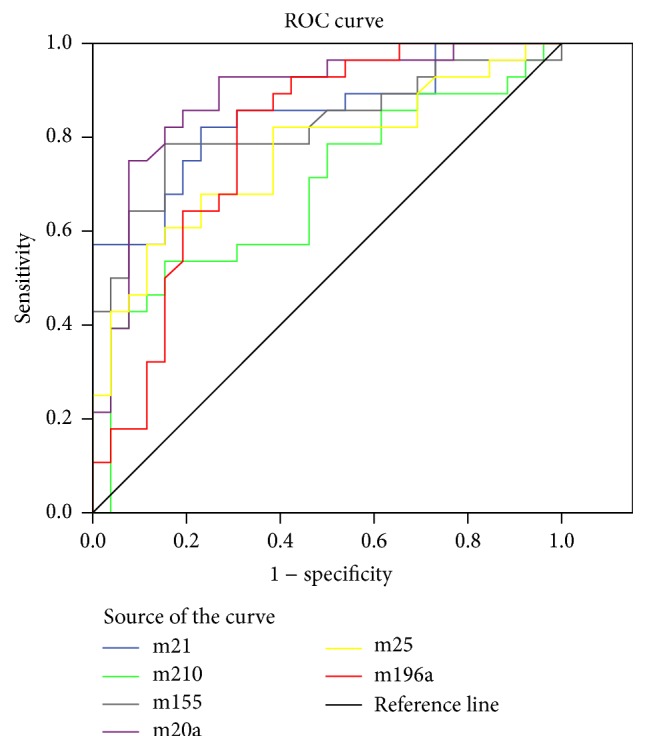
The ROC curves for the expression of the six plasma miRNAs and the corresponding calculated AUCs.

The expressions of plasma miRNA (miR-21, miR-210, miR-155, miR-196a, miR-20a, and miR-25) in PDAC patients and healthy controls were detected and compared. The logarithm (base 10) of expression was considered as the evaluation level for its normal distribution. As shown in Table 2 and Figure 1, the expression of six miRNAs in PDAC patients was significantly higher than that in healthy individuals. ROC curves were illustrated in Figure 2. The AUC (area under the curve) was 0.845 for miR-21 [95% CI, 0.740–0.949], 0.687 for miR-210 (95% CI, 0.543–0.831), 0.822 for miR-155 (95% CI, 0.707–0.937), 0.791 for miR-196a (95% CI, 0.665–0.916), 0.884 for miR-20a (95% CI, 0.790–0.978), and 0.763 for miR-25 (95% CI, 0.635–0.891), respectively.

### 3.2. miR-196a and miR-210 Expression Reached Significance in Univariate Analysis for Survival Time of PDAC Patients

**Figure 3:**
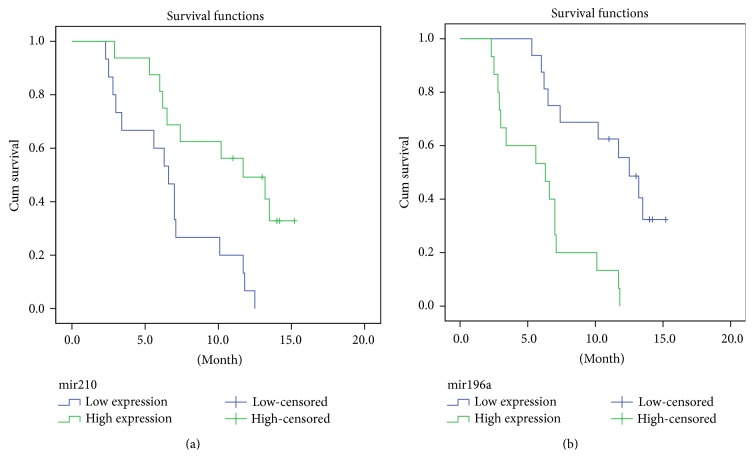
Kaplan-Meier overall survival curves for PDAC patients based on expression levels of the plasma miRNAs. The median logarithm value of the relative expression levels was used as a cut-off to classify patients into high- and low-expression miRNA groups. (a) and (b) stand for Kaplan-Meier overall survival curves for miR-210 and miR-196a, respectively.

Prognoses were performed in December 2013, obtaining available data from 31 cases by telephonic follow-up. The overall median survival time was 7.1 months, ranging from 2.3 to 15.2 months. Kaplan-Meier survival curves were constructed and compared by the log-rank test. Factors such as surgical method, lymph node metastasis, serosal infiltration, and comprehensive therapy were largely involved in the survival of the patient (Table 2). Based on the logarithm of absolute quantitative miRNA expression, the median values were taken as a cut-off to catalogue patients into high- and low-miRNA groups, respectively. The median survival times of the two groups for each miRNA were compared. A median survival of 6.3 months (95% CI, 2.3–10.3) was obtained in the high miR-196a group which was significantly shorter than that of 12.5 months (95% CI, 10.0–15.0) in the low miR-196a group (*P* = 0.001). Furthermore, high miR-210 expression with a median survival of 11.7 months (95% CI, 6.8–16.5) was considerably correlated with an improved survival in comparison to the low miR-210 expression with a median survival of 6.6 months (95% CI, 5.3–7.9) (*P* = 0.003). There were no significant differences in terms of the survival times (*P* = 0.714, 0.822, 0.394, and 0.362, resp.) between the high- and low-expression groups for the other four miRNAs (miR-21, miR-155, miR-20a, and miR-25) (Tables 3 and 4, Figure 3).

### 3.3. miR-210 Expression, miR-196a Expression, Lymph Node Metastasis, and Comprehensive Therapy Were Independent Factors for Survival Time of PDAC Patients by Multivariate Analysis

By Cox regression analysis, multivariate survival analysis was investigated for all 6 variables with significant *P* values in univariate analysis, such as surgical method, lymph node metastasis, serosal infiltration, comprehensive therapy, and expression of miR-196a and miR-210.  Table 5 showed that the overall survival time was significantly dependent on miR-210 expression, miR-196a expression, lymph node metastasis, and comprehensive therapy (*P* = 0.021, 0.013, 0.009, and 0.045, resp.). Therefore, miR-196a and miR-210 could be regarded as negative and positive prognostic markers, respectively.

## 4. Discussion

Facing the serious therapeutic and prognostic considerations of pancreatic adenocarcinoma, it is necessary to thoroughly investigate the expression of plasma microRNAs so as to establish their diagnostic and prognostic value for this condition. In this study, we have further confirmed the diagnostic value of six plasma miRNAs (miR-21, miR-210, miR-155, miR-196a, miR-20a, and miR-25), all of which were expressed at significantly higher levels in PDAC patients than in controls, with the *P* values in the range of 0.000 to 0.013 and the AUDs of 0.845, 0.687, 0.822, 0.791, 0.884, and 0.763, respectively. More importantly, we convincingly showed that plasma miR-196a overexpression was significantly associated with poorer survival rate. On the contrary, plasma miR-210 overexpression was observed to demonstrate significantly prolonged survival in PDAC patients. All these indications were based on the multivariate analysis with confounding clinical factors.

In addition to pancreatic cancer, the overexpression of miR-21 has been detected and correlated with poor survival in numerous cancers, such as glioma [12], breast cancer [13], colorectal cancer [14], gastric cancer [15], and prostate cancer [10, 16]. Thus overexpression of such miRNA may lack specificity and sensitivity. MiR-21 has been believed to function as an oncogenic miRNA. Some of miR-21-targeted genes or proteins have been investigated in previous studies. MiR-21 has been shown to affect cell proliferation, invasion, and the clinical outcome by downregulating the expression of tumor suppressors, such as PDCD4 [17, 18], TIMP3 [18], RECK [19], and KRIT1 [20]. The targeting of some other proteins, including Bcl-2, maspin, and PTEN, was also confirmed [6, 21]. Besides, overexpression of miR-21 could be involved in the inhibition of apoptosis as well as the acquisition of invasive properties [22–24]. MiR-155 has been reported to be an important diagnostic biomarker for PDAC as it could perform its oncogenic function by repressing proapoptotic tumor proteins [25] and regulate the invasion and migration of pancreatic cancer cells by modulating the STAT3 signaling pathway and by reducing SOCS1 expression [26]. Furthermore, the expression of MLH1 [27] and tumor suppressor SEL1L [28] appeared to be miR-155-modulating targets for the oncogenic function. Additionally, miR-155 was observed to indicate early pancreatic neoplasia [29, 30]. As for the prognosis, some studies demonstrated that high expression of miR-155 in tissues was correlated with poor survival [5–7]. However, the prognostic value of plasma miR-21 and miR-155 was not found in our study. This was probably explained as a result of the differences between the tissue and the plasma miRNAs expression levels. Alternatively, the limited sample size in this study may also be a reason.

High expression of miR-20a (the miR-17-92 cluster) has been reported in the plasma and tissue samples of patients with colon [31, 32], gastric [33, 34], prostate [35], and cervical cancer [36, 37]. In addition, miR-20a was upregulated at the different stages during nasopharyngeal carcinoma (NPC) progression. The expression level of such miRNA was reversibly associated with the prognosis of NPC [38]. The upregulation of miR-20a was suggested to enhance the metastatic potential of osteosarcoma [39] and breast cancer cells [40]. Interestingly, miR-20a was found to act as a tumor suppressor in breast cancer [41, 42] and inhibit the proliferation and invasion of pancreatic cells by negatively regulating STAT-3 protein expression [43] due to its possible double-sided role in tumors. MiR-25 (the miR-106b-25 cluster) is another double-sided miRNA in tumors. It could accelerate tumor growth and apoptosis by targeting Bim in gastric [44] and liver cancers [45]. Also, it appeared to promote ESCC cell migration and invasion both by overexpressing in tissues and by suppressing DH1 expression [46]. On the other hand, downregulated miR-25 was found in human colon cancer tissues, in which it inhibited cell proliferation and migration in cell lines and suppressed the growth of colon cancer cell xenografts by targeting Smad7 in colon cancer [47]. Therefore, we speculated that the genetic function of miR-20a and miR-25 may be tissue-specific. To date, the roles of miR-20a and miR-25 in the prognosis of pancreatic cancer have not been reported. In this study, there were no evidence to ascertain the expression of miR-20a and miR-25 with respect to survival time in PDAC patients. Thus, more in-depth studies need to be carried out in order to confirm their prognostic potential in PDAC.

Some previous studies evaluated tissue or plasma miR-196a during the survival of PDAC patients [48, 49]. Our study further confirmed that high expression of plasma miR-196a was an independent factor informative about survival time (*P* = 0.013). MiR-196a has been well believed to be associated with abnormal apoptosis, invasion, and proliferation of pancreatic cancer cells by downregulating ING5 expression [50]. In addition, miR-196a was also observed to be significantly deregulated in PanIN II-III, the early stage of PDAC [51]. A number of studies have also been conducted in the relationship between the expression of miR-196a and other malignancies [52, 53].

This is the first study, in the prognosis of PDAC patients, to demonstrate that plasma miR-210 was an independent positive factor based on multivariate analyses (*P* = 0.021). Despite the possible controversy caused by the contradiction between the diagnostic and prognostic value, our observation was consistent with studies regarding the diagnosis and prognosis of other malignancies such as non-small cell lung cancer [54], renal cell carcinoma [55], and soft-tissue sarcoma [56].

MiR-210 expression was indicated to regulate downstream genes, possibly by ERK- and PI3K/Akt-dependent signaling pathways [57] in the presence of a relative miR-210 upregulation in cardiomyocytes [58] and in adipose-derived stem cells [59]. Several studies found that miR-210 may function as a tumor suppressor by inhibiting ovarian cancer cell proliferation [60, 61]. Disruption of iron homeostasis in cancer cells could be a possible mechanism by which miR-210 constrained cancer cell proliferation [62]. Although miR-210 expression can induce cell migration [57], it did not exhibit any changes in the proliferation of Panc-1 cell lines [63]. MiR-210 was suggested to be able to regulate normoxic gene expression involved in tumor initiation and repress the initiation of tumor growth [64]. This might explain the mechanism of its positive impact on the prognosis of PDAC patients in this study. Greither et al. [7] reported that miR-210 tissue expression reversely correlated with survival time in PDAC patients. The sample origin, however, was tissue, not plasma. Moreover, further validation was performed in a breast cancer cell line (MCF-7) or tissue, neck cancer tissue to confirm that miR-210 was an independent negative prognostic factor for breast cancer and neck cancer patients [65, 66]. And also a meta-analysis of hazard ratio (HR) [67] was performed to evaluate the prognostic role of the miR-210 in different tumors including 9 published studies dealing with various carcinomas suggested that higher expression of miR-210 correlated with worse survival time, especially in breast cancer, and all of the samples are from tissues and most of the samples are from breast cancer patients.

The different prognostic value of miR-210 may be illustrated by the hypoxic environment it exerts the function and miR-210 having different regulatory effects on different cancer type and sample origin, plasm, or tissue. Therefore, the overall balance between pro- and antitumorgenesis genes differs in the specific tumor. Taken together, intensive studies by using large sample sizes are needed to investigate the paired-expression of miRNAs in both the tissue and the plasma for a variety of malignancies.

In summary, our study provided corroborative evidence to support the diagnostic value of six plasma miRNAs (miR-21, miR-210, miR-155, miR-196a, miR-20a, and miR-25) in PDAC patients. Significantly, miR-210 displayed a positive value in terms of prognosis and miR-196a was negative for prognosis. This observation may contribute to the clinical guidance. Furthermore, in-depth studies are needed to illuminate the molecular mechanisms and the therapeutic target(s) of miRNAs in PDAC patients.

## Figures and Tables

**Table 1:** Demographic data of human pancreatic ductal adenocarcinoma patients (PaC) and healthy control individuals.

Variable	PaC	Control	*P* value
Gender			
Male	20	18	0. 985
Female	11	10	
Age (years)	48.82 ± 9.75	44.65 ± 6.85	0.155
Cigarette smoking			
Yes	10	11	0.713
No	21	28	
Alcohol consumption			0.306
Yes	14	9	
No	17	19	

**Table 2:** Differential expression of six miRNAs in the plasma of the PDAC and the control groups.

	PDAC (lg copies/*μ*L)	Control (lg copies/*μ*L)	*P* value
miR-21	6.34 ± 0.44	5.65 ± 0.69	0.000
miR-210	4.94 ± 0.43	4.70 ± 0.39	0.013
miR-155	3.76 ± 0.74	3.26 ± 0.30	0.002
miR-196a	3.47 ± 0.44	2.82 ± 0.75	0.000
miR-20a	6.79 ± 0.44	5.50 ± 0.53	0.000
miR-25	6.61 ± 0.37	6.26 ± 0.33	0.000

**Table 3:** Univariate survival analysis of conventional prognostic factors based on the characteristics of the PDAC patients corresponding to the survival time.

Characteristic	Number	Median survival (month)	*P* value
Gender			0.550
Male	20	7.0	
Female	11	8.7	
Age (years)			0.521
≥60	17	10.7	
<60	14	6.9	
Cigarette smoking			0.255
Yes	10	6.5	
No	21	7.5	
Alcohol consumption			0.154
Yes	10	7.0	
No	21	8.1	
Abdominal pain			0.341
Yes	13	6.8	
No	18	8.1	
Jaundice			0.058
Yes	9	6.9	
No	22	9.9	
Tumor location			0.531
Head and neck	26	6.1	
Body and tail	5	8.8	
Operation method			0.009
Radical surgery	9	11.2	
Palliative surgery	17	6.3	
No surgery	5	5.5	
Tumor size (cm)			0.289
≤2	4	9.3	
>2–≤4	18	10.1	
>4	9	6.7	
Lymph node metastasis			0.001
Yes	19	5.0	
No	12	12.5	
Serosal infiltration			0.002
Yes	20	7.0	
No	11	13.2	
Vessel infiltration			0.053
Yes	17	6.8	
No	14	9.5	
Comprehensive therapy			0.024
Yes	19	10.4	
No	12	6.9	
Distant metastasis			0.079
Yes	6	6.8	
No	25	8.1	

**Table 4:** Univariate survival analysis of prognostic factors based on miRNA expression.

	MST of lower expression (month)	MST of higher expression (month)	*P* value
miR-21	7.0	7.4	0.714
miR-210	6.6	11.7	0.003
miR-155	7.0	7.1	0.822
miR-196a	12.5	6.3	0.001
miR-20a	11.7	7.0	0.394
miR-25	6.3	10.1	0.362

**Table 5:** Multivariate analysis of survival time of patients with PDAC.

Covariant	*B* coefficient	SE	*P* value	95% confidence interval
Lymph node metastasis	−0.198	0.231	0.009	0.098–1.212
Comprehensive therapy	1.211	0.545	0.045	0.055–2.051
miR-210	0.545	0.594	0.021	0.633–1.098
miR-196a	−1.255	0.051	0.013	0.923–1.055
