# Corrigendum to “Evaluation of Plasma MicroRNAs as Diagnostic and Prognostic Biomarkers in Pancreatic Adenocarcinoma: miR-196a and miR-210 Could Be Negative and Positive Prognostic Markers, Respectively”

**DOI:** 10.1155/2018/3782512

**Published:** 2018-09-06

**Authors:** Qi Yu, Changqing Xu, Wei Yuan, Chengfeng Wang, Ping Zhao, Lianzhen Chen, Jie Ma

**Affiliations:** ^1^Gastrointestinal Rehabilitation Center, Beijing Rehabilitation Hospital, Capital Medical University, Beijing 100144, China; ^2^Department of Digestive Medicine, Qianfoshan Hospital, Shandong University, 16766 Jingshi Road, Jinan 250014, China; ^3^State Key Laboratory of Molecular Oncology, National Cancer Center/Cancer Hospital, Chinese Academy of Medical Sciences and Peking Union Medical College, Beijing 100021, China; ^4^Department of Abdominal Surgery, Cancer Hospital of Chinese Academy of Medical Sciences, Peking Union Medical College, Beijing, China; ^5^Department of Pharmacy, National Cancer Center/Cancer Hospital, Chinese Academy of Medical Sciences and Peking Union Medical College, Beijing 100021, China; ^6^Department of Biotherapy, Beijing Hospital, National Center of Gerontology, Beijing 100730, China

In the article titled “Evaluation of Plasma MicroRNAs as Diagnostic and Prognostic Biomarkers in Pancreatic Adenocarcinoma: miR-196a and miR-210 Could Be Negative and Positive Prognostic Markers, Respectively” [1], there were errors in the authors' affiliations. The correct affiliation list is shown above.
